# Enzymatic production of defined chitosan oligomers with a specific pattern of acetylation using a combination of chitin oligosaccharide deacetylases

**DOI:** 10.1038/srep08716

**Published:** 2015-03-03

**Authors:** Stefanie Nicole Hamer, Stefan Cord-Landwehr, Xevi Biarnés, Antoni Planas, Hendrik Waegeman, Bruno Maria Moerschbacher, Stephan Kolkenbrock

**Affiliations:** 1 Institute of Plant Biology and Biotechnology, Westphalian Wilhelm's-University Münster, Schlossplatz 8, 48143 Münster, Germany; 2 Laboratory of Biochemistry, Institut Químic de Sarrià (IQS), Universitat Ramon Llull (URL), Via Augusta 390, E-08017 Barcelona, Spain; 3 Bio Base Europe Pilot Plant, Rodenhuizekaai 1, 9042 Ghent, Belgium

## Abstract

Chitin and chitosan oligomers have diverse biological activities with potentially valuable applications in fields like medicine, cosmetics, or agriculture. These properties may depend not only on the degrees of polymerization and acetylation, but also on a specific pattern of acetylation (PA) that cannot be controlled when the oligomers are produced by chemical hydrolysis. To determine the influence of the PA on the biological activities, defined chitosan oligomers in sufficient amounts are needed. Chitosan oligomers with specific PA can be produced by enzymatic deacetylation of chitin oligomers, but the diversity is limited by the low number of chitin deacetylases available. We have produced specific chitosan oligomers which are deacetylated at the first two units starting from the non-reducing end by the combined use of two different chitin deacetylases, namely NodB from *Rhizobium* sp. GRH2 that deacetylates the first unit and COD from *Vibrio cholerae* that deacetylates the second unit starting from the non-reducing end. Both chitin deacetylases accept the product of each other resulting in production of chitosan oligomers with a novel and defined PA. When extended to further chitin deacetylases, this approach has the potential to yield a large range of novel chitosan oligomers with a fully defined architecture.

Chitin, a β-(1, 4)-linked polymer composed of *N*-acetyl-d-glucosamine (GlcNAc, a) residues, is one of the most abundant biopolymers on earth, occurring e.g. in the exoskeleton of crustaceans and insects, and in the cell walls of fungi. Chitosan is the completely or partially deaceylated form of chitin, thus being composed of GlcNAc as well as d-glucosamine (GlcN, d) units. Chitosan polymers as well as oligomers of chitin and chitosan show diverse biological activities and, therefore, have a broad range of different potential applications in various fields, including medicine, cosmetics, food, and agriculture^1^,^2^,^3^,^4^.

Although chitin occurs in a wide variety of species, most of today's commercially available chitin and its derivatives are produced from shrimp or crab shell waste through a combination of chemo-physical treatments^5^. These chitosans can be produced with different degrees of polymerization (DP) and different degrees of acetylation (DA), but their pattern of acetylation (PA) is invariably random^6^. As chitin polymers are insoluble in aqueous solutions and chitosan polymers are soluble only at slightly acidic pH values, interest has shifted lately towards the more soluble chitin and chitosan oligomers^2^. Biological activities of chitosan oligomers have been shown or are hypothesized to be strongly dependent on the DP, DA, and PA^7^,^8^,^9^. To verify this hypothesis, defined chitosan oligomers with fully known architecture are needed. Production of chitosan oligomers by partial chemical or physical depolymerisation of the respective polymers, as typically done today, has severe disadvantages. Not only does the production of the oligomers typically involve harsh thermo-chemical treatments or strong physical forces, which may be environmentally unfriendly and/or partially destructive to the oligosaccharides produced, but also the production is highly difficult to control leading to broad heterogeneous mixtures, and the outcome is strongly dependent on the chemical and physical characteristics of the starting material^10^. Partial enzymatic hydrolysis of chitosan polymers using chitosan hydrolysing enzymes such as chitinases or chitosanases with well-defined cleavage specificities has been proposed as an alternative to chemical or physical depolymerisation^11^,^12^,^13^. But, this attempt is also strongly dependent on the starting material and it, too, leads to the production of heterogeneous mixtures of chitosan oligomers. Still, due to the cleavage specificities of the enzymes, the resulting mixture will be better defined than the chitosan oligomer mixtures obtained by chemical or physical depolymerisation^2^,^12^. A fully controlled method potentially leading to a broad range of fully defined products is chemical synthesis of chitosan oligomers from monomeric building blocks. However, this attempt is time and labour intensive and the yields are rather low^14^,^15^. Alternatively, chitin oligomers can be efficiently converted into defined chitosan oligomers by the help of specific chitin deacetylases (EC 3.5.1.41) and chitin oligosaccharide deacetylases (EC 3.5.1.-)^9^,^16^. A number of different chitin deacetylases from different sources have been described^17^,^18^,^19^,^20^,^21^,^22^. Among these, two highly interesting candidates for the production of fully defined chitosan oligomers are the highly specific chitin oligosaccharide deacetylases NodB from *Rhizobium* spp. and COD from *Vibrio cholerae*. NodB deacetylates exclusively the GlcNAc unit at the non-reducing end, whereas COD deacetylates the second unit from the non-reducing end^18^,^20^,^23^. The limitation of this technique lies in the rather low number of recombinant chitin deacetylases available today with a known and fully defined mode of action, leading to a very limited number of defined chitosan oligosaccharides that can be obtained in this way.

To at least partially overcome this limitation, we have expressed *cod* from *V. cholerae* as well as *nod*B from *Rhizobium* sp. GRH2 in *Escherichia coli* and purified the recombinant enzymes. Starting with chitin oligomers of defined DP (a_n_), we used the single enzymes for the *in vitro* generation of two different, fully defined mono-deacetylated chitosan oligomers (da_(n-1)_ and ada_(n-2)_). As a general agreement the first letter of the oligomers mentioned here represents the residue at the non-reducing end, while the last one represents that of the reducing end. As a proof-of-principle study, we then combined the two deacetylases in a single reaction and generated the expected doubly deacetylated oligomers (dda_(n-2)_), showing that this approach opens a way to produce novel chitosan oligomers with hitherto unreported PA.

## Results and Discussion

### Isolation and analysis of *Rhizobium* sp. GRH2 *nod*B

We designed consensus-degenerate hybrid oligonucleotide primers (CODEHOP)^24^ based on the sequences of multiple *nod*B genes from different *Rhizobium* strains, and used these to identify and subsequently clone the chitin deacetylase gene from *Rhizobium* sp. GRH2. The analysis of 16S rDNA sequences by BLASTn revealed that *Rhizobium* sp. GRH2 is most closely related to *Rhizobium leguminosarum* bv. *trifolii* (99% identity).

### Production of enzymes in *E. coli* and characterization of purified enzymes

**Figure 1:**
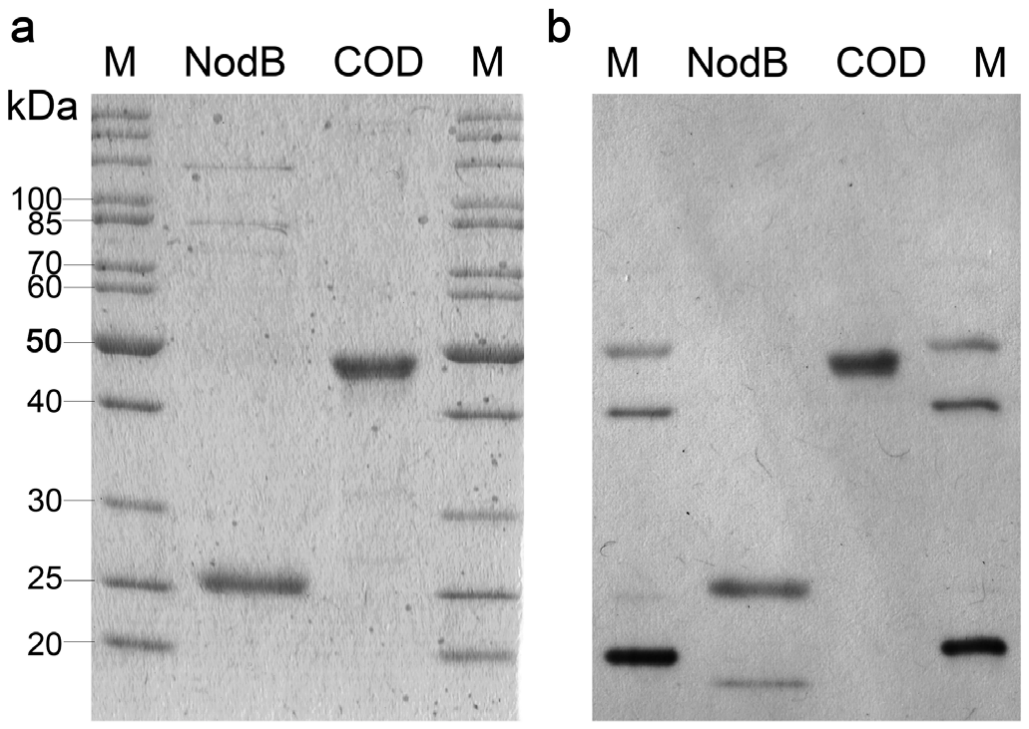
SDS-PAGE and corresponding western blot of recombinant NodB from *Rhizobium* sp. GRH2 and COD from *Vibrio cholerae* purified from the crude extract of *E. coli* BL21 (DE3) [pET-22b(+)::*nod*B_StrepIIC] and *E. coli* Rosetta 2 (DE3) [pLysSRARE2, pET-22b(+)::*cod*_StrepIIC], respectively. The enzymes were purified by streptactin affinity chromatography and 3 μg of each enzyme was applied to the gel. Enzymes were visualized either by staining with ethyl violet/zincon (a) or by enhanced chemiluminescence using HRP-coupled streptactin conjugate after western blotting (b). M: peqGOLD Protein-Marker II (Peqlab, Erlangen, Germany).

The GRH2 *nod*B gene was expressed in *E. coli* BL21 (DE3), whereas the *cod* gene was expressed in *E. coli* Rosetta 2 (DE3) [pLysSRARE2] essentially as previously reported^23^,^25^. Both genes contained a downstream located Strep-tag II encoding sequence for the purification by streptactin affinity chromatography. Sodium dodecyl sulfate-polyacrylamide gel electrophoresis (SDS-PAGE) and western blot analysis revealed single bands for both enzymes, with apparent molecular masses close to the expected ones of 24.4 and 45.5 kDa for NodB and COD, respectively (Fig. 1).

The chitin deacetylase activity of NodB was tested against GlcNAc_1–6_. The hydrolysis products were characterized by ultra high performance liquid chromatography - evaporative light scattering detection - electrospray ionization - mass spectrometry (UHPLC-ELSD-ESI-MS), revealing that NodB was not active towards GlcNAc_1_, but converted GlcNAc_2–6_ completely into mono-deacetylated chitosan oligomers (Table 1). To our knowledge, this is the first report of obtaining enzymatically active, recombinant NodB without the need of refolding it from insoluble inclusion bodies^20^. COD from *V. cholerae* is active on short chitin oligosaccharides^18^,^25^, and we tested COD with GlcNAc_1–6_ under the same conditions and analysed the hydrolysis products in the same way as we did for NodB. As expected, COD converted GlcNAc_2–6_ completely into mono-deacetylated chitosan oligomers, but not GlcNAc_1_ (Table 1). The optimal pH for NodB was 9 and the optimal hydrolysis temperature was 37°C (Supplementary Fig. S1 online) as tested with GlcNAc_5_. According to literature, the optimal pH of COD is 8 and the optimal hydrolysis temperature is 45°C^18^. For COD no unspecific hydrolysis products were detected after prelonged incubation, as opposed to NodB, where negligible amounts of double deacetylated chitosan oligomers were detected after prolonged incubation at high enzyme concentrations.

**Figure 2:**
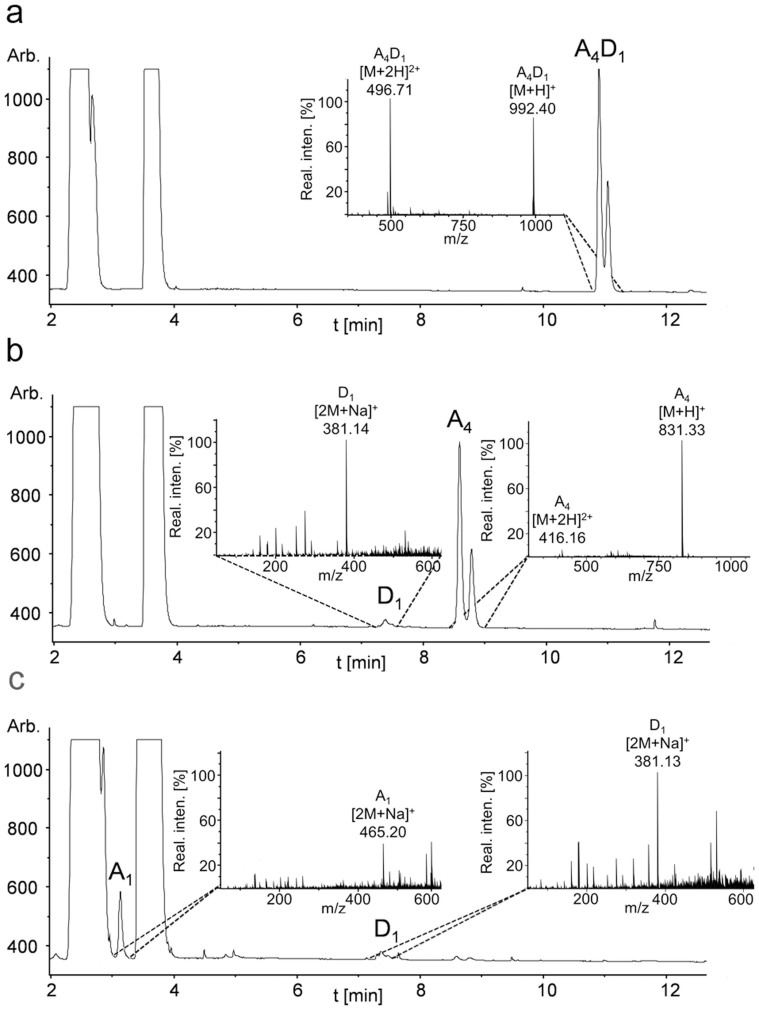
Determination of the pattern of acetylation (PA) of GlcNAc_5_ (a_5_) after hydrolysis with NodB from *Rhizobium* sp. GRH2 by enzymatic sequencing in combination with UHPLC-ELSD-ESI-MS analysis. To determine the PA of the exclusively mono-deacetylated chitosan pentamer (a_4_d_1_) as generated by NodB (a), it was subsequently incubated with the GlmA_TK_ GlcNase from *Thermococcus kodakaraensis* KOD1. This enzyme exclusively cleaves GlcN units from the non-reducing end leading to GlcNAc_4_ (a_4_) and GlcN_1_ (d_1_) (b). In the next step GlmA_TK_ was replaced by the GlcNAcase *Bs*NagZ from *Bacillus subtilis* 168, which exclusively removes terminal GlcNAc units, starting from the non-reducing end. The reaction yielded GlcNAc_1_ (a_1_) and GlcN_1_ (d_1_) monomers (c). This approach revealed the pattern of acetylation of a chitin pentamer after incubation with NodB from *Rhizobium* sp. GRH2 to be daaaa.

Enzymatic sequencing^23^ in combination with UHPLC-ELSD-ESI-MS analysis showed that recombinant NodB deacetylated the first unit from the non-reducing end generating da_1–5_ chitosan-oligomers (Figure 2), which is in agreement with previous reports on recombinant NodB, refolded from the pellet fraction^20^. Likewise, the same sequencing methodology showed that COD deacetylated the second unit from the non-reducing end generating ada_1–3_ chitosan-oligomers, in agreement with previous reports^18^.

### Production and analysis of defined chitosan oligomers

**Figure 3:**
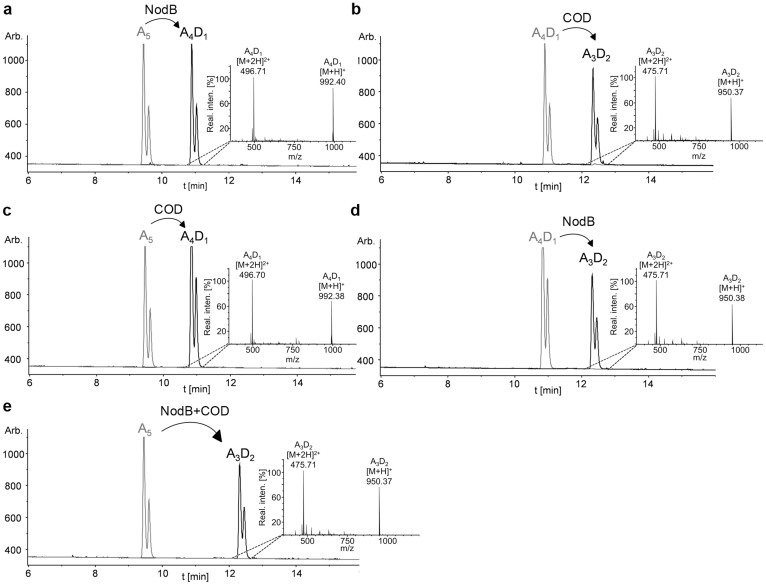
UHPLC-ELSD-ESI-MS analysis of *in vitro* combinations of NodB and COD. The substrate GlcNAc_5_ (a_5_) was deacetylated with NodB (a). NodB was removed and the obtained mono-deacetylated chitosan pentamer (a_4_d_1_) was further deacetylated with COD (b). The same was done *vice versa*: GlcNAc_5_ (a_5_) was deacetylated with COD (c) in the first step, and the enzyme was then replaced by NodB (d). Furthermore, NodB and COD were combined in a single reaction leading to a double-deacetylated chitosan pentamer in one reaction (e). All reactions were carried out in ammonium hydrogen carbonate buffer (pH 8) at 37°C for 2 h.

**Figure 4:**
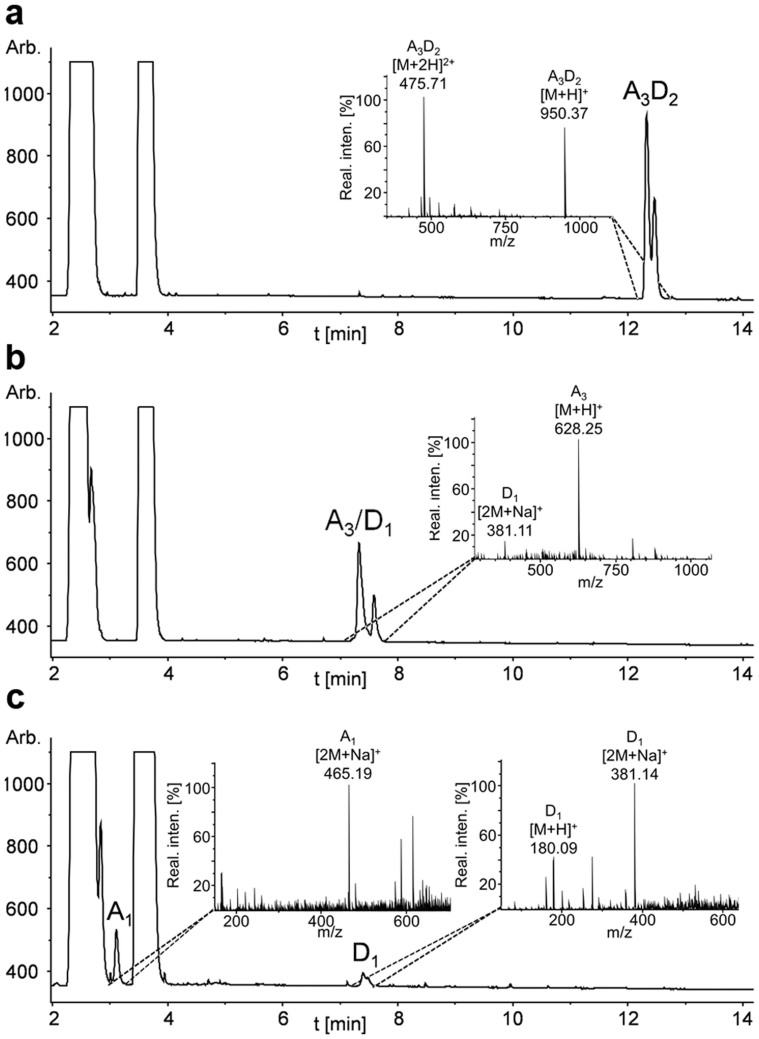
Enzymatic sequencing in combination with UHPLC-ELSD-ESI-MS analysis of double-deacetylated chitosan pentamer (a_3_d_2_). The product a_3_d_2_ was obtained after the incubation of GlcNAc_5_ (a_5_) with NodB and COD (a). To determine the PA, it was first hydrolysed with the GlcNase GlmA_TK_, which removes exclusively GlcN units from the non-reducing end. The reaction resulted in GlcN_1_ (d_1_) and GlcNAc_3_ (a_3_) (b). In the next step, GlmA_TK_ was replaced by the GlcNAcase *Bs*NagZ, which exclusively removes GlcNAc units from the non-reducing end, resulting in GlcN and GlcNAc monomers (c).The enzymatic sequencing of the simultaneous hydrolysis product of NodB and COD revealed that the first two units starting from the non-reducing end were deacetylated, giving a specific chitosan oligomer with the novel PA ddaaa.

To test whether the chitin oligosaccharide deacetylases NodB and COD are also active on partially deacetylated chitosan oligomers – and whether the enzymes can be used in combination to broaden the spectrum of defined chitosan oligomers with a specific PA - we deacetylated GlcNAc_5_ with NodB, removed NodB, and used the mono-deacetylated chitosan pentamer as a substrate for COD. The same was done in reverse order, and in a combined reaction of NodB and COD together. In all cases, GlcNAc_5_ was completely converted into doubly deacetylated chitosan pentamers (Figure 3). The PA of the doubly deacetylated chitosan pentamer was determined using enzymatic/mass spectrometric sequencing which revealed that the first two units from the non-reducing end were deacetylated in the combined as well as in both sequential reactions of NodB and COD (Figure 4). In addition of GlcNAc_5_, also GlcNAc_2–4_ and GlcNAc_6_ were converted into the respective doubly deacetylated chitosan oligomers in a combined reaction of NodB and COD (Table 2).

**Figure 5:**
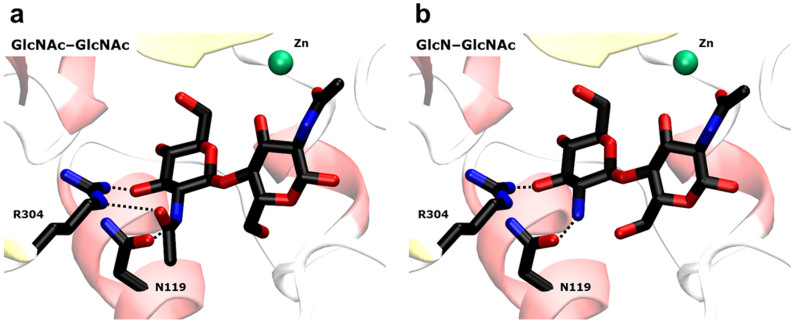
Structures of AA and DA substrates in the active site of COD. A) X-ray structure of the complex COD·AA^25^. B) Modelled structure with the DA substrate. Ligands are shown as thick lines, metal ion as a green sphere. Only the amino acids interacting with the N-acetyl group at the non-reducing end are shown (as thick lines). The removal of this acetyl group does not alter significantly the interactions map at the active-site, where only one hydrogen-bond with R304 is lost.

In addition to the *in vitro* experiments, binding of partially deacetylated chitosan dimers and trimers to COD, was examined *in silico*. The recently solved three dimensional structures of COD in complex with GlcNAc_2_, and GlcNAc_3_^25^ were used as reference structures (PDB accession codes: 4NZ1 and 4OUI respectively). The dimeric and trimeric substrates where manually converted to all possible deacetylated compounds (i.e., da, ad, dd, daa, ada, aad, dda, dad, add, ddd). Docking calculations were done with flexible ligands inside a search-space grid-box that covers the whole active site. Figure 5 shows the crystal structure of COD in complex with the disaccharide substrate aa, and the modelled structure of the complex with the NodB product da, which properly binds to form a competent complex. The closest amino acid residues to the 2-NAc or 2-NH_2_ in the glucosyl unit on the non-reducing end are Arg304 and Asn119. Removal of the acetyl group in the da substrate does not alter significantly the interactions map where only one hydrogen-bond with Arg304 is lost. The binding affinity of all acetylated, partially deacetylated, and fully deacetylated compounds to COD was calculated by means of the VINA scoring function^26^ (Table 3). Not surprisingly, the fully acetylated compounds are the ones with strongest binding affinity towards COD (−9.4 kcal mol^−1^ for aa, −10.6 kcal mol^−1^ for aaa). On the other hand, the deacetylated compounds produced by COD (ad and ada) loose affinity towards the enzyme (increase in energy of roughly 1 kcal mol^−1^). Interestingly, the deacetylated compounds produced by NodB (da and daa) are able to bind COD with a stronger affinity than the COD originating products, but the interaction energy to COD is still reduced by 0.8 to 0.4 kcal mol^−1^ relative to the natural fully acetylated substrates.

In other words, the contribution to the binding affinity of the *N*-acetyl substituent of the sugar ring at the non-reducing end is only 0.8 kcal mol^−1^ for chitobiose in COD, in agreement with the lost of one hydrogen bond with Arg304 in the deacetylated substrate (Figure 5). This contribution is lower for chitotriose (just 0.5 kcal mol^−1^). It is expected that this contribution will be even lower for longer oligosaccharides. Thus, although COD is more active on aa and k_cat_ decreases with increasing oligosaccharide length^18^,^25^, compounds such as chitotetraose or chitopentaose continue to be substrates of COD even if they are deacetylated at the non-reducing end because the loss of affinity due to the acetyl substituent of the sugar ring at the non-reducing end will presumably be negligible.

The fact that NodB and COD accept the products of each other offers new possibilities for the biotechnological production of defined chitosan oligomers. Assuming that also other chitin deacetylases can accept partially deacetylated products as substrate, a rather large number of different fully defined chitosan oligomers with different PA could be produced by combining a rather limited number of different chitin deacetylases.

**Figure 6:**
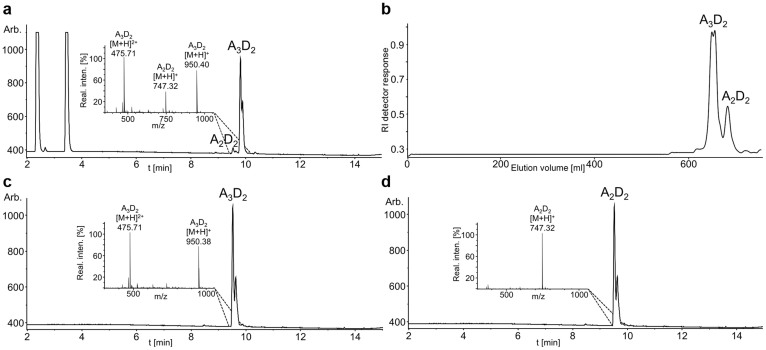
Production and UHPLC-ELSD-ESI-MS analysis of doubly deacetylated chitosan tetramers and pentamers at mg-scale. Doubly deacetylated chitosan tetramers (A_2_D_2_) and pentamers (A_3_D_2_) were produced by deacetylating biotechnologically produced chitin pentamer and tetramer with NodB and COD in a combined reaction overnight (a). The resulting doubly deacetylated chitosan oligomers A_3_D_2_ and A_2_D_2_ were purified and separated from each other using SEC (b). Fractions containing A_3_D_2_ (c) and A_2_D_2_ (d) were pooled separately and analysed using UHPLC-ELSD-ESI-MS.

In order to produce sufficient amounts of specifically mono as well as doubly deacetylated chitosan oligomers for further downstream bio-testing, we biotechnologically produced chitin pentamer in *E. coli*, of which we then deacetylated 100 mg in a combined reaction using NodB and COD at mg-scale. The chitosan pentamers thus obtained were purified using size-exclusion chromatography (SEC), separating them from chitosan tetramers, which originated from a by-product of the recombinant production of chitin pentamers in *E. coli*. The purified products were analysed using UHPLC-ELSD-ESI-MS and proved to be highly pure (Figure 6). We obtained 27 mg of highly pure doubly deacetylated chitosan pentamer (ddaaa) and 4 mg of equally pure doubly deacetylated chitosan tetramer (ddaa).

As this method can be scaled up, it is feasible to produce fully defined chitosan oligomers in sufficient amounts for trial applications in different fields such as cosmetics or bio-medicine. Evaluating the scientific and commercial potential of fully defined chitosan oligomers is not possible at the moment due to the lack of well-defined chitosan oligomers on the market. When the chitin oligomers used as a substrate are of biotechnological origin, as in this study, our method has the added advantage that the chitosan oligomers produced, can be guaranteed to be free of allergenic contaminants.

## Methods

### Bacterial strains, vectors and culture conditions

*E. coli* strains TOP10 (Invitrogen, Darmstadt, Germany) and DH5α^27^ were used for general cloning, whereas Rosetta 2 (DE3) [pLysSRARE2] and BL21 (DE3) (Novagen, Darmstadt, Germany) were used for protein expression. The vector pCRII-TOPO (Invitrogen) was used for cloning of PCR fragments, whereas pET-22b(+) (Novagen, Darmstadt, Germany) including a StrepII tag sequence upstream of the multiple cloning site (MCS)^23^ was used for cloning and expression. *E. coli* was grown on LB agar, in LB medium, or in auto-induction medium^28^. Media were supplemented with the appropriate antibiotics (34 μg ml^−1^ chloramphenicol and/or 100 μg ml^−1^ ampicillin).

### Preparation of genomic DNA from *Rhizobium* sp. GRH2 and 16S rDNA analysis

Genomic DNA was extracted from *Rhizobium* sp. GRH2 as described by Rainey *et al.*^29^. The 16s rDNA was amplified with Phusion Hot Start II High Fidelity proof reading DNA Polymerase (Fisher Scientific, Schwerte, Germany) using three different primer pair combinations (27F/1525R, 27F/926R and 16S-F/16S-Rev). All primer sequences are listed in Supplementary Table S1. Only 27F/926R yielded a product, which was purified, sequenced and analysed using BLASTn.

### Identification of unknown *nod*B gene from GRH2 and plasmid construction

Consensus-degenerate hybrid oligonucleotide primers (CODEHOP) [24] were designed against known *nod*B genes from different *Rhizobium* strains as previously described. All primer sequences used during the study are listed in Supplementary Table S1. The genes were amplified using Mango-Taq DNA Polymerase (Bioline, Luckenwalde, Germany) and different combinations of CODEHOPs (B-for, D-rev, C-for, E-rev). Obtained PCR products were cloned in pCRII-TOPO for sequencing, followed by sequence analysis using BLASTx and alignment with CloneManager (Scientific & Educational Software, Cary, USA). Inverse PCR was used to isolate the upstream and downstream regions from genomic DNA. Total DNA was each digested with either *Sac*I or *Hin*dIII and circularized with rapid T4 DNA ligase.

The circulized DNA was used as template for a PCR with different inverse primer combinations. Amplified PCR products were ligated into pCRII-TOPO vector for sequencing. Promising sequences were aligned with CloneManager to generate a contig. The full-length *nod*B gene was isolated by standard PCR using a further CODEHOP primer (BC-for) designed for known *nod*B genes from different *Rhizobium* strains and specific primers that bind within the partially identified regions of the *nod*C gene from *Rhizobium* sp. GRH2, which is located upstream of *nod*B. In the last step the full length *nod*B gene was amplified by using specific primers and Phusion Hot Start II High Fidelity proof reading DNA Polymerase (Fisher Scientific) to exclude possible mutations, which may had occurred during the different PCR amplification steps with the non-proof reading Mango-tag polymerase.

### Plasmid construction

The *nod*B gene from *Rhizobium* sp. GRH2 was synthesized by GenScript (New York, USA) and inserted into the vector pET-22b(+)StrepIIC after digestion with *Nde*I and *Sac*I. The construction of pET-22b(+)StrepIIC, pET-22b(+)::*cod*_StrepIIC for overexpression of *cod* from *V. cholerae* (GenBank: AAF94439.1), and pET-22b(+)::*glm*A_TK__StrepIIC for overexpression of *glm*A_TK_ from *T. kodakaraensis* KOD1 (GenBank: BAD85943.1) was described earlier^23^. To improve the expression rate of *nag*Z from *B. subtilis* 168 (GenBank: CAB11942.1) in *E. coli*, the gene was codon optimized and synthesized by GenScript with a StrepII encoding sequence downstream of the gene and inserted into pET-22b(+)::StrepIIC after digestion with *Nde*I and *Sac*I.

### Synthesis and purification of enzymes

*E. coli* BL21 (DE3) harbouring either pET-22b(+)::*nod*B_StrepIIC or pET-22b(+)::*nag*Z_StrepIIN+C and *E. coli* Rosetta 2 (DE3) [pLysSRARE2] harbouring either pET-22b(+)::*cod*_StrepIIC or pET22-b(+)::*glm*A_TK__StrepIIC were cultivated in autoinduction medium. *E. coli* BL21 (DE3) [pET-22b(+)::*nod*B_StrepIIC] was cultivated at 37°C for 4 h and subsequently at 18°C for 40 h, whereas the other strains where incubated at 26°C for 48 h. The enzymes were purified as described earlier^23^. The protein concentration was determined with Pierce™ BCA Protein Assay Kit (Pierce Biotechnology, Rockford, USA).

### SDS-PAGE and western blot analysis

Protein samples were heated in SDS loading buffer (8% (w/v) SDS in 0.25 M Tris/HCl pH 6.8, 20% glycerol (v/v), 0.4% (w/v) bromophenol blue, 0.4% (v/v) β-mercaptoethanol) at 99°C for 10 min and separated by SDS-PAGE in a 12% (w/v) gel^30^. Separated proteins were visualized with zincon/ethyl violet staining^31^ or transferred to a nitrocellulose membrane (GE Healthcare Europe GmbH, Freiburg, Germany) using a semi-dry transfer procedure^32^. Strep-tag II fusion proteins were detected with Strep-Tactin-horseradish peroxidase (HRP) conjugate and chemiluminescent reaction was developed according to the instruction of the manufacturer (IBA GmbH, Göttingen, Germany).

### Determination of pH and temperature optimum for NodB

The pH and temperature optima of NodB were determined indirectly, by measuring the amount of acetate released during the enzymatic reaction, using an acetic acid assay kit (R-bioharm AG, Darmstadt, Germany) adapted for microtiter plates. The pH optimum was determined over the pH range 4–12 at 37°C for 2 h, in buffer containing 100 mM NH_4_HCO_3_, 20 mM TEA, 20 mM KH_2_PO_4_ and 20 mM Na_2_HPO_4_. Teorell and Stenhagen buffer (100 mM citric acid, 100 mM phosphoric and 100 mM boric acid^33^) was used for high-pH conditions, overlapping at pH 10. The temperature optimum was determined at 4°C, 22°C, 37°C, 45°C and 55°C at pH 9 using the buffer and conditions described above. The reaction volume was set to 400 μl, comprising 50 mM buffer, 1 mM chitin pentamer (Megazyme, Bray, Ireland) and 0.5 μM purified NodB. The reactions were stopped and the amount of released acetic acid was measured directly.

### UHPLC-ELSD-ESI-MS analysis of chitin and chitosan oligomers

The applied UPLC-ELSD-ESI-MS^n^ method was developed based on the methods described previously by Leijdekkers *et al*. and Remoroza *et al*.^34^,^35^. Analysis of chitin and chitosan oligomers were done using a Dionex Ultimate 3000RS UHPLC system (Thermo Scientific, Milford, USA) coupled to an evaporative light scattering detector (Model Sedex 90LT, Sedere, Alfortville Cedex, France) and an ESI-MS^n^-detector (amaZon speed, Bruker, Bremen, Germany). Chitin and chitosan oligomers were separated by hydrophilic interaction chromatography (HILIC) using an Acquity UPLC BEH Amide column (1.7 μm, 2.1 mm × 150 mm; Waters Corporation, Milford, MA, USA) in combination with a VanGuard pre-column (1.7 μm, 2.1 mm × 5 mm; Waters Corporation, Milford, USA). The flow rate was set to 0.4 ml min^−1^ and the column oven temperature to 35°C. Aliquots of 1 μl of sample were injected into the system using an autosampler. Samples were eluted from the column with a gradient of A and B. Eluent A was composed of 80:20 acetonitrile/water and eluent B of 20:80 acetonitrile/water. Both eluents contained 10 mM NH_4_HCO_2_ and 0.1% (v/v) formic acid. The separation of samples was done over 15 min using the following elution profile: 0–2.5 min, isocratic 100% A; 2.5–12.5 min, linear from 0% to 75% (v/v) B; followed by column re-equilibration: 12.5–13.5 min linear from 75% to 0% (v/v) B, isocratic 100% (v/v) A from 13.5–15 min. A 1:1 splitter (Accurate, Dionex Corporation, Sunnyvale, CA, USA) was used to split the eluent to the ELSD and to the ESI-MS^n^ detector. The gas pressure of the ELSD HPLC nebulizer was set to 3.5 bar, the gas flow to 1.75 l min^−1^, the evaporation temperature to 50°C and the gain to 12. MS-detection was performed in a positive mode with the capillary voltage set to 4 kV, the end plate offset voltage to 500 V, the pressure of the nebulizer to 15 psi, the flow rate of the dry gas to 8 l min^−1^ and the dry temperature to 200°C. Mass spectra were acquired over a scan range from m/z 50–2000 using enhanced resolution scan mode and were analysed using Data Analysis 4.1 software (Bruker, Bremen, Germany). Precision of the masses according to the instrument specifications are ±0.15 m/z units.

### Analysis of enzyme activity of NodB and COD

Substrate specificity of NodB and COD was tested towards GlcNAc_1–6_. GlcNAc_1_ was purchased from Sigma Aldrich (München, Germany) whereas GlcNAc_2–6_ was purchased from Megazyme (Bray, Ireland). GlcNAc_1–6_ (1 mM) were incubated with either 2.5 μM NodB or 2.5 μM COD in 50 mM NH_4_HCO_3_ (pH 8) at 37°C overnight. To ensure that the hydrolysis activity was caused by the activity of NodB or COD and not by an endogenous *E. coli* enzyme, GlcNAc_5_ was in parallel incubated under the same conditions as describe above with 3 μl crude extract (4 mg ml^−1^) of *E. coli* Rosetta2 (DE3) [pLysSRARE2] harbouring either pET22-b(+)::*nod*B_StrepIIC, pET22-b(+)::*cod*_StrepIIC or the empty vector control. The reaction volume was set to 20 μl. All products were analysed by UHPLC-ELSD-ESI-MS.

### Determination of the PA of the generated chitosan oligomers

The PA of the different chitosan oligomers generated by NodB and COD, or by a combined action of both enzymes was determined by enzymatic sequencing^23^. Samples were analysed by UHPLC-ELSD-ESI-MS analysis instead of HPTLC.

The composition of the shake flask and minimal batch fermenter medium was the same as described by Waegeman *et al*.^36^. Fed-batch medium consisted of 500 g l^−1^ glucose, 1 g l^−1^ MgSO_4_×7H_2_O and 30 g l^−1^ NH_4_Cl. All media were supplied with 0.1 g l^−1^ ampicillin. The recombinant *E. coli* strain was pre-cultured in two shake flasks of 2 l total volume, filled with approximately 0.2 l shake flask medium and grown at 30°C while constantly shaking at 200 rpm. After 24 hours, 100 ml of broth was transferred into two shake flasks of 5 l total volume filled with 2 l shake flask medium at 30°C while constantly shaking at 200 rpm. After 24 hours the resulting fermentation broth (4 l in total) was inoculated into a 150 l fermenter (Sartorius, Göttingen, Germany). Fermentation conditions were; temperature: 30°C, stirrer speed: minimum 500 rpm and if pO_2_ drops below 30% stepwise increased, aeration: 10 slpm, pH: 7, pressure: 500 mbar. Feeding was started when glucose was depleted in the batch phase and the feed rate applied was 4 l h^−1^. The total fermentation time was 110 h. After fermentation, the broth was harvested and cells were separated from supernatant by ceramic tangential flow microfiltration (Tami Industries, Nyons, France, 0.45 μm). Both the cell and supernatant fraction contained the fully acetylated pentamer and were further purified. Cells were disrupted by a homogeniser (GEA Process Engineering, Mechelen, Belgium, 10 l h^−1^) and cell debris was removed by tangential flow microfiltration (Kleenpak, Pall, Zaventem, Belgium, 0.45 μm). In both fractions, salts were removed by ion exchange (Amberlite, Dow, Tessenderlo, Belgium) and the solutions were further concentrated by wiped film evaporation (Carl Canzler, Germany, 150 l h^−1^, 60°C, 70 mbar) and finally spray-dried (Xedev, Zelzate, Belgium). After purification, 371 g of product was obtained, which consisted for 85% of fully acetalyated pentamer (GlcNAc_5_).

Purified chitin pentamer (100 mg) was incubated with 0.2 μM NodB and 0.6 μM COD in 100 ml 50 mM NH_4_HCO_3_ buffer (pH 8) at 37°C overnight. The sample was freeze dried and solved in water before size exclusion chromatography (SEC). After SEC the sample was diluted with water and freeze dried. The obtained product was analysed using UHPLC-ELSD-ESI-MS.

### Size-exclusion chromatography (SEC)

Deacetylated chitosan oligomers were purified on three HiLoad 26/600 Superdex 30 pg columns (GE Healthcare Europe GmbH, Freiburg, Germany) in a row with an overall dimension of 2.60 × 180 cm. Ammonium acetate buffer (0.15 M, pH 4.5) was used as mobile phase and the flow rate was set to 0.8 ml min^−1^. The effluent was monitored with an online refractive index detector (1260 Infinity Refractive Index Detector, Agilent Technologies Deutschland GmbH, Böblingen, Germany) which was coupled to a datalogger^13^,^37^. Fractions containing oligomers were pooled and analysed by UHPLC-ELSD-ESI-MS.

### Calculation of relative binding affinities of partially deacetylated chitobiose and chitotriose to COD structure

Three dimensional structures of COD in complex with chitobiose and chitotriose have been recently obtained^25^ and deposited in the Protein Data Bank with accession codes 4NZ1 and 4OUI respectively. Polar hydrogens were added to the receptor protein structure with AutoDockTools^38^. AutoDock4.2 atom typing was used. Gaisteger partial charges were computed for each atom with AutoDockTools. Ligand structures (chitobiose, aa and chitotriose, aaa) were extracted from the corresponding PDB files. For each ligand, a series of partially and fully deacetylated compounds were generated by removing the corresponding *N*-acetyl group with AutoDockTools while keeping the overall geometry of the molecules. Thus, three dimensional structures aa, ad, da, dd, aaa, ada, aad, add, dad, ddd were obtained. Every ligand was parametrized in the same way as the receptor. Each ligand was docked onto COD structure by means of AutoDock VINA algorithm^26^. A grid-box of 30 × 26 × 30 A^3^ centered at the active site was used as the search space for docking. Interaction energies between ligands and COD receptor were calculated with VINA scoring function^26^. Reported values in Table 3 are an estimation of the differences in free energy upon binding for those docking poses in which the ligand binds in a productive orientation at the active site. The uncertainty of these calculations is 0.6 kcal mol^−1^. This was estimated as the average range of VINA score values obtained for a series of similar docking poses (structures within 1.5 A of the lowest score one).

## Author Contributions

S.N.H., S.C.-L., X.B., A.P., H.W., B.M.M. and S.K. designed the experiments and discussed the results. S.N.H., S.C.-L., X.B. and H.W. performed the experiments. S.N.H. wrote the main manuscript text to which X.B. and H.W. contributed. S.N.H. prepared all figures and tables except for table 3 and figure 5 which were prepared by X.B. All authors reviewed and revised the manuscript.

## Supplementary Material

Supplementary Information Supplementary Material

## Figures and Tables

**Table 1:** Determination of substrate specificity of NodB and COD by UHPLC-ELSD-ESI-MS. GlcNAc_1–6_ (1 mM) was incubated with 2.5 μM NodB or COD in ammonium formate buffer (pH 9) or ammonium hydrogen carbonate buffer (pH 8), respectively, at 37°C overnight

	expected products		determined products NodB		determined products COD	
Subst.	M_w_ A_1–6_	M_w_ A_0–5_D_1_	m/z	M_w_	m/z	M_w_
A_1_	221.09	179.08	A_1_ (465.14 [2M + Na]^+^)	221.07	A_1_ (465.14 [2M + Na]^+^)	221.07
A_2_	424.17	382.16	A_1_D_1_ (383.16 [M + H]^+^)	382.16	A_1_D_1_ (383.14 [M + H]^+^)	382.16
A_3_	627.25	585.24	A_2_D_1_ (586.24 [M + H]^+^)	585.24	A_2_D_1_ (586.23 [M + H]^+^)	585.23
A_4_	830.33	788.32	A_3_D_1_ (789.35 [M + H]^+^)	788.35	A_3_D_1_ (789.35 [M + H]^+^)	788.35
A_5_	1033.41	991.40	A_4_D_1_ (496.70 [M + H]^2+^)	991.40	A_4_D_1_ (496.69 [M + H]^2+^)	991.38
A_6_	1236.49	1194.48	A_5_D_1_ (598.24 [M + H]^2+^)	1194.48	A_5_D_1_ (598.23 [M + H]^2+^)	1194.48

**Table 2:** Analysis of double deacetylation of GlcNAc_2–6_ by a combined action of NodB and COD, analysed using UHPLC-ELSD-ESI-MS. GlcNAc_2–6_ (1 mM) was incubated with 2.5 μM of both NodB and COD in ammonium hydrogen carbonate buffer (pH 8) at 37°C overnight

	expected products			determined products NodB + COD	
Subst.	M_w_ A_2–6_	M_w_ A_1–5_D_1_	M_w_ A_0–4_D_2_	m/z	M_w_
A_2_	424.17	382.16	340.15	A_0_D_2_ (341.16 [M + H]^+^)	340.15
A_3_	627.25	585.24	543.23	A_1_D_2_ (544.27 [M + H]^+^)	543.27
A_4_	830.33	788.32	746.31	A_2_D_2_ (747.35 [M + H]^+^)	746.35
A_5_	1033.41	991.40	949.39	A_3_D_2_ (475.73 [M + H]^2+^)	949.46
A_6_	1236.49	1194.48	1152.47	A_4_D_2_ (577.28 [M + H]^2+^)	1152.56

**Table 3:** Calculated binding affinities of partially and fully deacetylated chitobiose and chitotriose to COD structure. * natural products of COD reaction

Chitobiose-like compound	Binding Affinity (kcal mol^−1^)	Chitotriose-like compound	Binding Affinity (kcal mol^−1^)
AA	−9.4	AAA	−10.6
DA	−8.7	DAA	−9.9
AD*	−8.1	ADA*	−9.5
DD	−7.2	DDD	−8.2
		AAD	−10.1
		DDA	−8.8
		ADD	−9.1
		DAD	−9.4
