# Supplementary material for: Enzymatic production of defined chitosan oligomers with a specific pattern of acetylation using a combination of chitin oligosaccharide deacetylases
Source: Sci Rep. 2015 Mar 3;5:8716. doi: 10.1038/srep08716 (PMC4346795; doi:10.1038/srep08716)
Supplement: Supplementary Information — Supplementary Material [file srep08716-s1.doc]

**Enzymatic production of defined chitosan oligomers with a specific pattern of acetylation using a combination of chitin oligosaccharide deacetylases**

Stefanie Nicole Hamer^1^, Stefan Cord-Landwehr^1^, Xevi Biarnés^2^, Antoni Planas^2^, Hendrik Waegeman^3^, Bruno Maria Moerschbacher^1^, Stephan Kolkenbrock^1^ *

^1^Institute of Plant Biology and Biotechnology, Westphalian Wilhelm’s-University Münster, Schlossplatz 8, 48143 Münster, Germany

²Laboratory of Biochemistry, Institut Químic de Sarrià (IQS), Universitat Ramon Llull (URL), Via Augusta 390, E-08017 Barcelona, Spain

^3^ Bio Base Europe Pilot Plant, Rodenhuizekaai 1, 9042 Ghent, Belgium

^*^ S. Kolkenbrock, evocatal GmbH, Alfred-Nobel-Str. 10, 40789 Monheim am Rhein, Germany, S.Kolkenbrock@evocatal.com, Phone: +49(0)2173-40994-20, Fax: +49(0)2173-40994-40

**Supplementary Material**

**Primer used during the study**

Supplementary Table S1 Primer used during the study

| **Primer** | **Oligonucleotide sequences (5′–3′)** | **Type** |
| --- | --- | --- |
| B-for | 5'-GCGAGCTGCAGGTCTATGTCrtngaygaygg-3' | CODEHOP primers |
| D-rev | 5'-GGCGCACGG Gccrcarcarca-3' |
| C-for | 5'-CCTCCAACCGCAACGACwsntggytnac-3' |
| E-rev | 5'-CAGCATCAG GATGGTCAGAtgnckrtcytc-3' |
| BC-for | 5'-CCGTCGTCCGCCATATCmgngcnccnta-3' |
| B (inverse) rev | 5‘-ACGCTAGTGTCGAATCCGAGTC-3‘ | Inverse primers |
| C (inverse) for | 5‘-ATTGACCGCTAGCAACCGAAACG-3‘ |
| E (inverse) for | 5‘-GTCGCAATTTTTCCGGGGCAAG-3‘ |
| GRH2 rev | 5‘-CCTCGCGAAGCAGAATGAAGTC-3‘ | Specific primers |
| GRH 2 SRP | 5’-ATAAAGGCACTGTCGCTGTC-3’ |
| GRH 2 SRP II | 5’-GATACGGGCCCATCCTATCC-3’ |
| NodB for | 5‘-CATGCGTCTCACATGCATATGACGCATTTTGACTGCTTATC-3‘ | Full gene length primer |
| NodB rev | 5‘-CCGCTCGAGTTAGAGCTCCTTTGTTAAGGGATGTTGAGGAAG-3‘ |
| 27f | 5‘-AGAGTTTGATCMTGGCTCAG-3‘ | 16S rDNA  primer |
| 1525R | 5‘-AAGGAGGTGATCCAGCC-3‘ |
| 926R | 5‘-CCGTCAATTCCTTTRAGTTT-3‘ |
| 16S-FW | 5‘-GAAGAGTTTGATCATGGCTCAG-3‘ |
| 16S-Rev | 5‘-ACGACAGCCATGCAGCACCT-3‘ |

**pH and temperature optimum of NodB**


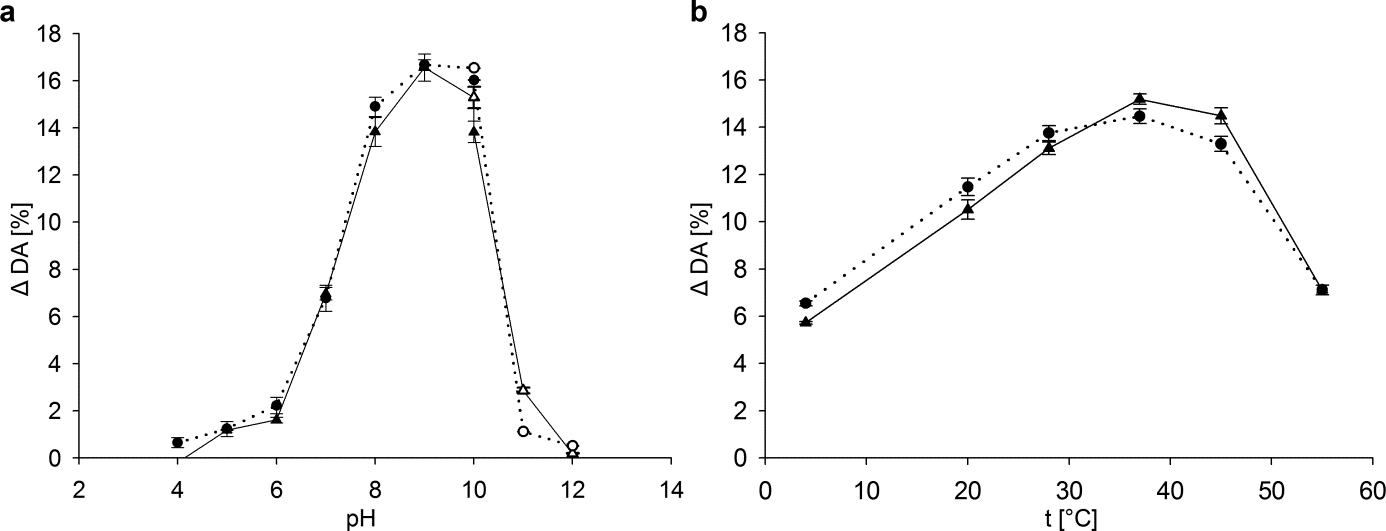


**Supplementary Figure S1** pH optimum **(A)** and temperature optimum **(B)** of NodB. The pH and temperature optima were determined by measuring the amount of acetate released during the enzymatic reaction. Based on these values, the ΔDA [%] was calculated. The measurements were done twice using two independently prepared enzyme batches (circles, triangles).The pH optimum was determined by the incubation of the enzyme at pH 4-12 at 37°C for 2 h in buffer containing 100 mM NH_4_HCO_2_, 20 mM TEA, 20 mM KH_2_PO_4_ and 20 mM Na_2_HPO_4_ (●,▲). Teorell and Stenhagen buffer (100 mM citric acid, 100 mM phosphoric and 100 mM boric acid) was used for high-pH conditions, overlapping at pH 10 (○, Δ). The temperature optimum was determined at pH 9 for 2 h using the first buffer mentioned above.
